# Therapeutic hypothermia attenuates tissue damage and cytokine expression after traumatic brain injury by inhibiting necroptosis in the rat

**DOI:** 10.1038/srep24547

**Published:** 2016-04-15

**Authors:** Tao Liu, Dong-xu Zhao, Hua Cui, Lei Chen, Ying-hui Bao, Yong Wang, Ji-yao Jiang

**Affiliations:** 1 Department of Neurosurgery, Ren Ji Hospital, School of Medicine, Shanghai Jiao Tong University, Shanghai, China; 2 Department of Neurological Srgery, The People’ s Hospital of Pu Dong New Area, Shanghai, China

## Abstract

Necroptosis has been shown as an alternative form of cell death in many diseases, but the detailed mechanisms of the neuron loss after traumatic brain injury (TBI) in rodents remain unclear. To investigate whether necroptosis is induced after TBI and gets involved in the neuroprotecton of therapeutic hypothermia on the TBI, we observed the pathological and biochemical change of the necroptosis in the fluid percussion brain injury (FPI) model of the rats. We found that receptor-interacting protein (RIP) 1 and 3, and mixed lineage kinase domain-like protein (MLKL), the critical downstream mediators of necroptosis recently identified *in vivo*, as well as HMGB1 and the pro-inflammation cytokines TNF-α, IL-6 and IL-18, were increased at an early phase (6 h) in cortex after TBI. Posttraumatic hypothermia (33 °C) led to the decreases in the necroptosis regulators, inflammatory factors and brain tissue damage in rats compared with normothermia-treated TBI animals. Immunohistochemistry studies showed that posttraumatic hypothermia also decreased the necroptosis-associated proteins staining in injured cortex and hippocampal CA1. Therefore, we conclude that the RIP1/RIP3-MLKL-mediated necroptosis occurs after experimental TBI and therapeutic hypothermia may protect the injured central nervous system from tissue damage and the inflammatory responses by targeting the necroptosis signaling after TBI.

Traumatic brain injury (TBI) is disastrous to patients in clinic. When the brain contusion happens, we can do less to prevent the proceeding and substantial cascade of cell death, inflammation, edema and immune response, which finally results in functional disorder and even death^1^. Cell death, one of the essential parts directly related to the behavior and memory function in the central nervous system (CNS), also plays a key role in the maintenance of tissue homeostasis throughout life in multicellular organisms. Therefore, the strategy of targeting the inhibition of cell death process may contribute to TBI therapy^2^,^3^.

For many years, apoptosis had been the most extensively characterized form of regulated cell death that mediated by caspases, whereas necrosis has been only seen as an unregulated passive cell death process. However, base on the genetic and biochemical evidence and the specific chemical inhibitors of necrosis screened out, this process has been redefined as a molecularly controlled form of cell death, which is termed necroptosis for its unique mechanism^4^,^5^. Classically, the necroptosis is initiated by TNF signaling pathway, which has been reported to get involved in neurobehavioral and histologic outcomes after TBI by genetically engineered mice^6^,^7^. TNF-α is identified as a pleiotropic cytokine, binding to the TNF receptor (TNFR) 1 or 2. Ligand-bound TNFR1 is immediately internalized, leading to formation of a cytosolic death-inducing signalling complex (DISC), which plays a pivotal role in a variety of cellular responses, including differentiation, proliferation, inflammatory cytokine production, death and survival^8^. When the process of necroptosis is initiated, receptor-interacting protein (RIP) 1 (together with its cognate kinase RIP3) is recruited to form a supramolecular complex included TNFR-associated death domain (TRADD), FAS-associated protein with a death domain (FADD) and caspase-8, which are better known as necrosome and considered as the executed platform of necroptosis^9^. The formation of necrosome is highly regulated by the mutual phosphorylation of the RIP1 and RIP3^10^. RIP1 is a serine/threonine kinase containing a RIP homotypic interaction motif (RHIM) that binds to the RHIM in RIP3. Although the role of RIP1 in necroptosis has been extensively study, evidence suggests that activation of RIP3 can induce necroptosis independent of RIP1^11^ and that RHIM-dependent autophosphorylation of RIPK3 results in the recruitment and phosphorylation of mixed lineage kinase domain-like (MLKL)^12^,^13^, which contributes to a conformational change in the pseudokinase domain and finally leads to the exposure of cells by changing permeabilization in plasm membrane^14^,^15^. Stimulation of TNFR family was known to mediate apoptosis in most cells before, but it is reported by recent studies that apoptosis is at least partially able to shift to necrosis under conditions deficient for caspase-8 or treated with the pan-caspase inhibitor Z-VAD-fmk^16^,^17^. Necrostatin-1 (Nec-1), a potent and efficient inhibitor of necroptosis^4^, is demonstrated to decrease brain tissue damage and inflammation, suppress autophagy and apoptosis, and improved behavior performance after TBI in rodents^18^,^19^, but it is not proof for necroptosis^20^. However, in the absence of a specific molecular marker, the only definite criterion for necroptosis has been dependence on RIPK3 and MLKL^5^, whether this mode of cell death involved in pathology of TBI still needs further study. Since necroptosis has been reported to play a key role in many diseases^4^,^17^,^21^,^22^, it may be an important therapeutic target for future investigations.

Posttraumatic moderate hypothermia has been reported by several researches to improve behavioral and histopathological outcomes^23^,^24^,^25^,^26^, and it appears to exert neuroprotective effects against traumatic and ischemic brain injury by decreasing apoptosis and autophagy occurred in neuronal cells by modulating glia activation, cytokines release, ion channel change and excitotoxicity etc.^2^,^3^,^23^,^27^,^28^. Here, we tested the hypothesis that necroptosis contributes to cell death after traumatic brain injury (TBI) and that therapeutic hypothermia treatment would inhibit the process after fluid percussion injury (FPI) in rat. Therapeutic hypothermia reduced the expression of pivotal regulator of necroptosis, short-term tissue damage, and HMGB1 as well as the cytokines TNF-α, IL-6 and IL-18 in rat subjected to FPI. The data suggest an important role for necroptosis in the pathogenesis of TBI, and improve the understanding of the mechanisims of therapeutic hypothermia for patients with head injury.

## Materials and Methods

### Animals

All animal procedures were approved by the Animal Care and Experiment Committee of the School of Medicine, Shanghai JiaoTong University and all experimental procedures were performed in accordance with the guideline of the National Institutes of Health on animal care and the ethical guidelines for study of pain^29^. Male Sprague–Dawley rats (210 to 260 g) were maintained for at least 7 days before the study in an air-conditioned room with a constant temperature (25 °C), and a 12-h light/dark cycle, free access to food and water.

### Traumatic Brain Injury

Animals groups were randomly divided as follows: SHAM (n = 30), TBI-Normothermia-6 hours (h) (TBI, n = 18), TBI-Normothermia-24 h (n = 12), TBI-Normothermia-72 h (n = 12), TBI-DMSO-6 h (n = 6), TBI-Nec-1–6 h (n = 6), TBI-GSK′ 872-6 h (n = 6), TBI-NSA-6 h (n = 6) and TBI-Hypothermia-6 h (HT, n = 12). In this study, rats underwent moderate head injury ranging from 1.8 to 2.2 atm in a fluid percussion injury (FPI) device (Virginia Commonwealth University Biomedical Engineering, Richmond, VA, USA) as described by our laboratory before^30^. Briefly, the animals were anesthetized with a nitrous oxide/oxygen mixture (70%/30%) containing 2% halothane. Tracheal intubation and monitoring of blood pressure and blood gas analysis were carried out. Then the animals were placed in a stereotactic frame and the scalp was incised sagittally. A 4.8- mm-diameter circular craniotomy was drilled 2 mm to the left of the midline between the bregma and the lambda. A Luer-Lok (Becton Dickinson, Mountain View, CA) needle hub was placed in the skull hole and fixed with dental cement. After the cement dried, rats were connected to the FPI device by the hub, and then the metal pendulum was dropped down. A small volume of saline was dropped on the exposed brain surface to protect the injured tissure. Meanwhile, blood pressure and arterial blood gases were monitored by placing catheters within the tail artery. The brain temperature was estimated with a thermistor probe inserted into the temporalis muscle, and the body temperature was also measured with a rectal probe. The temperature was maintained at a constant level of ~37 °C.

Hypothermia was performed as described in a previous study (Jiang *et al.*) and initiated 30 minutes after TBI. Briefly, a brain temperature of 33 °C was achieved within 15 minutes by wrapping the body of anesthetized rats with plastic bag filled ice and water for a period of 4 h and followed by a slow rewarming period (over 90 minutes). The normothermic groups were maintained at 37 °C throughout the procedure. Following these procedures, the animals were returned to their home cages and free to food and water.

### Drug preparation and administration

The specific inhibitors of necroptosis Nec-1 (Selleck, Houston, TX, USA), GSK′ 872 (Biovision, Mountain View, CA, USA) and necrosulfonamide (NSA) (TRC, Toronto, Canada) were diluted with DMSO to a final concentration of 25 mM. Following FPI, the animal was placed into a stereotaxic frame and a 23-gauge stainless steel guide cannula attached to a 25-μL Hamilton^®^ syringe was stereotactically inserted (coordinates: AP −0.8 mm, lateral 1.4 mm, 3.5 mm beneath the pial surface). By using a syringe pump, 6 μL of each compounds and dilute DMSO were injected at 30 min postinjury at a rate of 0.5 μL/min. These dose and method of administration were based on our pre-experiment. Following the intracerebroventricular (icv) injection, the animal was removed from the stereotaxic device, and maintained at a rectal temperature of 37 °C throughout surgery, recovery.

### Quantitative Real-Time PCR

The mRNA levels of the RIP3 and MLKL were analyzed in the injured rats and the sham controls at 6, 24, 72 hours after TBI. Six rats were studied at each time and followed the procedures previously described^30^. Briefly, tissue from the cerebral cortex was homogenized in Trizol reagent (Invitrogen, Carlsbad, CA, USA) for extraction of total RNA based on the manufacturer’s protocol. Total RNA was quantitated using the 8453 UV-visible spectroscopy system (Agilent Corporation, Palo Alto, CA, USA). Complementary DNA of 1 μg of total RNA was synthesized using PrimeScript^TM^ RT Master Mix (Perfect Real Time) (Takara Bio, Shiga, Japan). The mRNA levels in each sample were determined by real-time PCR using SYBR Green I Dye. The reverse transcription product was included in the SYBR Premix Ex Taq kit (Perfect Real Time) (Takara Bio), along with rat RIP3 and MLKL primers, and the mixture was placed in a real-time PCR thermal cycler (Lightcycler System; Roche Diagnostics Corp., Indianapolis, IN, USA). Thermal cycling parameters were 30 sec at 95 °C, followed by 40 cycles of 5 sec at 95 °C, 5 sec at 60 °C, and 30 sec at 72 °C. At the end of the program, melting curve analysis was performed at 72 °C for 30 sec, followed by a cooling step at 37 °C for 30 sec. Each sample was also run with primers for a housekeeping gene. The following primer pairs were used: rat RIP3 forward primer 5′-CTGTCGCCTGCTAGAGGAAG-3′ and reverse primer 5′-TCTGCTAACTTGGCGTGGAG-3′, rat MLKL forward primer 5′-CCCGAGTTGTTGCAGGAGAT-3′ and reverse primer 5′-TCTCCAAGATTCCATCCGCAG-3′, and β-actin forward primer 5′-AGGGAAATCGTGCGTGACAT-3′, and reverse primer 5′-TGGCCATCTCTTGCTCGAAG-3′. For relative comparison of each gene, we analyzed the Ct value with the ΔΔCt method normalizing.

### Immunoblotting

Tissue samples were snap-frozen in liquid nitrogen and stored at −80 °C before use. A 1 × 1 cm section of cortex or total hippocampus was homogenized in extracton buffer (20 mmol/L Tris- HCl, pH: 7.5, 150 mmol/L NaCl, 1% Triton X-100; 1 mmol/L ethylenediaminetetraacetic acid, 1 mmol/L ethyleneglycol- tetraacetic acid, 2.5 mmol/L pyrophosphate, 1 mmol/L b-glycerophosphate) containing protease inhibitor mixture (Roche Applied Science, Indianapolis, IN). Total protein concentration was measured by the BCA protein assay kit (Bio-Rad Laboratories, Hercules, CA, USA). Total protein of 20 μg was separated on a 15% SDS-polyacrylamide gel by electrophoresis. The proteins were transferred to PVDF immunoblotting membranes (Bio-Rad Laboratories, USA). The membranes were blocked with 5% non-fat milk in phosphate-buffered saline (PBS) with 0.1% Tween-20 and then incubated with primary antibodies at 4 °C overnight as follows: polyclonal antibody to RIP1 (1:2,000; Cell Signaling Technology, Beverly, MA, USA), RIP3 antibody (1:1000, ProSci Inc., Poway, CA, USA), HMGB1 antibody (1:1000, Cell Signaling Technology, Beverly, MA, USA), TNF-α antibody (1:1000, R&D Systems Inc., Minneapolis, MN, USA), IL-6 antibody (1:1000, R&D Systems Inc., Minneapolis, MN, USA), IL-18 antibody (1:1000, R&D Systems Inc., Minneapolis, MN, USA),rabbit anti-MLKL (1:400, Santa Cruz, Santa Cruz, CA, USA), anti-β-actin antibody (1:5,000; Sigma-Aldrich, St Louis, MO, USA), and appropriate secondary horseradish peroxidase (HRP)-linked antibodies for 1 h at room temperature. Visualization of signal was enhanced by chemiluminescence using a phototope-HRP detection kit (Pierce, Rockford, IL, USA). Quantification of band density was performed using the NIH Image J software (Bethesda, MD, USA), and data were normalized to β-actin.

### Immunohistochemistry

At 6 h after FPI, animals were anesthetized and perfused with 500 mL of 4% paraformaldehyde. The brains were quickly removed and postfixed in 4% paraformaldehyde at 4 °C for 20 h, then immersed in 30% sucrose in 0.1 M PBS at 4 °C overnight and subsequently embedded in paraffin. Sections with 4-μm thickness were deparaffinized and rehydrated in gradient alcohol. Slides were then placed in the microwaveable vessel filled with the Sodium Citrate Buffer (10 mM Sodium Citrate, 0.05% Tween 20, pH 6.0) for heat-induced epitope retrieval by a microwave. 2.5% Normal Horse Serum (ready-to-use) (Vector Laboratories, Burlingame, CA, USA) was used for blocking. Staining was performed by incubating the sections with anti-RIP1 (1:200, H207, Santa Cruz, USA) and anti-RIP3 (1:500, ProSci, USA) antibodies at 4 °C, overnight. After endogenous peroxidase activity was blocked with 0.3% H_2_O_2_ in TBS for 15 min, appropriate ImmPress anti-rabbit peroxidase (Vector Lab) was dropped onto the sections followed by a 1-hour incubation at room temperature. Signals were developed with 3,3′-diaminobenzidine (CST) for 2 min, and haematoxylin was applied for counterstain. For negative controls, sections were incubated with PBS instead of primary Ab. To assess quantitatively the numbers of immunopositive neurons on sections in the central of contusion area (corresponded to approximately 4.44 mm posterior to bregma), four microscopic fields of hippocampus and cortex around the contusion area each section were photographed randomly (200× magnifications, Nikon TE300; Nikon) and the numbers of positive neurons per high microscopic field were counted by an investigator who was blinded to the experimental groups. Data were normalized to the total cell number of the fields selected.

### Contusion volumes

For contusion volume assessment, tissue sections 10-μm thick were taken at 150-mm intervals as described by previous study^31^. Briefly, The cortical contusion volume was quantified by outlining the entire cortical contusion in sequential hematoxylin and eosin-stained sections (14–20 sections/animal, depending upon the contusion size) using a Nikon TE300 microscope (Olympus America, Center Valley, PA) at 12.5× magnification. The cortical contusion boundaries depend on the shades of H&E staining, which consisted of pyknotic neurons, reactive astrocytes, macrophage infiltration, and disordered white matter tracts. Contusion volume was calculated by numeric integration of sequential areas and analyzed in a blinded fashion. Images representing the section at bregma level −4.44 mm are shown.

### Statistics

Data were analyzed using GraphPad Prism Version 6.0c software and are expressed as mean ± standard deviation. For comparisons between multiple groups, the significance of differences in between-group means was tested by one-way analysis of variance (AOVA) combined with the Dunnett post-hoc test or Turkey-Kramer test. Homogeneity of variance was assessed by the F-test or the Brown-Fortsythe test. An unpaired t test was used to compare two groups. Nonparametric data were analyzed using the Kruskal-Wallis test or the Mann-Whitney U test as appropriate. P values of significance used were *P < 0.05.

## Results

### Physiological Parameters Were Stable During the Operation

Physiological parameters were within the normal ranges during the surgical period (Table 1). No significant differences were found between each group except for the comparisons of temperature among TBI-hypothermic, TBI-normothermic and as well as the sham group (p < 0.05).

### mRNA and Protein Levels of the RIP3 and MLKL Were Elevated After TBI

**Figure 1:**
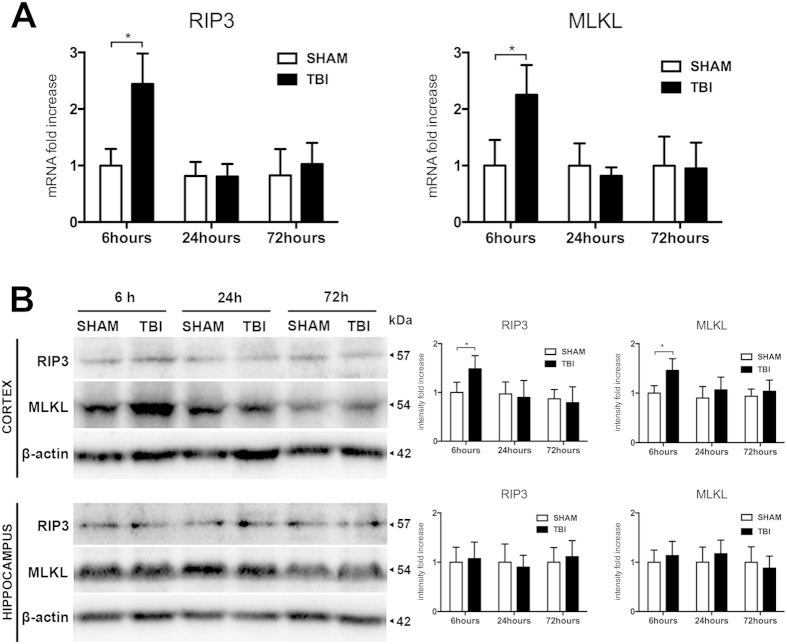
Temporal profile of RIP3 and MLKL mRNA and proteins expression after traumatic brain injury. Rats were sacrificed at the indicated time points after TBI. Total RNA and proteins were prepared from the ipsilateral cortex and hippocampus. (**A**) Quantitative real-time PCR analysis for RIP3 and MLKL at 6, 24 and 72 hours in cortex in sham-operated animals (SHAM) and normothermic group (TBI) after TBI (n = 6 each); (**B**) Western blot analysis for RIP3 and MLKL in cortex and hippocampus at 6, 24 and 72 hours after injury (n = 6 each). Lane-loading differences were normalized by levels of β-actin; Data are expressed as mean ± SD and analyzed using unpaired t test vs. SHAM. *P < 0.05.

In order to unravel a potential role of necroptosis in TBI, we first assessed the mRNA and protein levels of the RIP3 and MLKL in the brain. The variation in the RIP3 and MLKL mRNA was measured by quantitative RT-PCR in the ipsilateral cortex over time after TBI. As shown in Fig. 1, at 6 h after TBI, the levels of both the RIP3 and the MLKL mRNA were increased significantly compared with the sham group (p < 0.05). However, at 24 h and 72 h after TBI, the RIP3 and MLKL mRNA levels had no significant difference than the corresponding values in sham group (Fig. 1A). Western blot analysis confirmed that RIP3 and MLKL protein amounts were markedly increased at 6 h compared with each sham group (p < 0.05) in cortex but not at 24 h or 72 h after TBI, similar to the results of the RT-PCR above (Fig. 1B). In contrast, no statistical significant changes in RIP3 and MLKL protein levels were detected in the total hippocampus lysate at any of the time courses tested. These data suggest that RIP3/MLKL-mediated necroptosis may be involved in the neuron loss at 6 h but declined at 24 h and 72 h in cortex after TBI.

### Hypothermia Inhibited RIP1, RIP3 and MLKL expression in Ipsilateral Cortex After TBI

**Figure 2:**
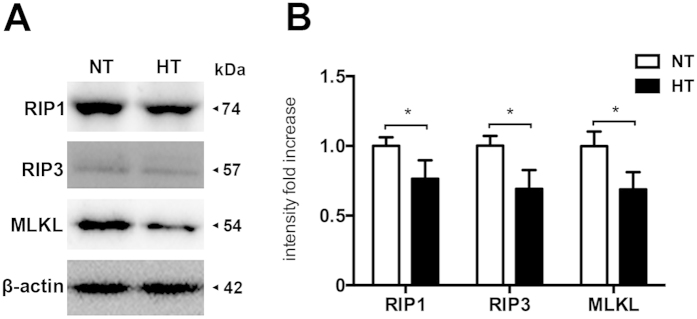
Hypothermia reduces necroptosis processing in the injured cortex after TBI. Rats were sacrificed at 6 h after TBI. Total proteins were prepared from the ipsilateral cortex of TBI-Normothermia (NT) and TBI-Hypothermia (HT). (**A**) Immunoblot analysis of RIP1, RIP3 and MLKL in lysates of ipsilateral cortex after injury (n = 6 each). (**B**) Quantitative analysis of RIP1, RIP3 and MLKL after TBI. Lane-loading differences were normalized by levels of β-actin; Data are expressed as mean ± SD; Data of RIP1 are analyzed using ANOVA with Dunnett’s post-hoc test vs. SHAM. *P < 0.05.

To determine the effects of hypothermia on necroptosis processing at 6 h after TBI, we performed immunoblot analysis of ipsilateral cortical lysates from sham-surgical animals, normothermic animals and hypothermic-treated animals. In addition, the effect of hypothermia on the protein levels of RIP1, a critical mediator of necroptosis^4^ and a potential target protein of apoptosis and autophagy^19^, was also tested in parallel. As shown in Fig. 2, TBI significantly increases RIP1 protein levels in ipsilateral cortex at 6 h after TBI (p < 0.05), whereas hypothermia following TBI remarkably reduced RIP1, RIP3 and MLKL (p < 0.05).

### Hypothermia Decreased RIP1 and RIP3 Immunoreactivity in Cortical and Hippocampal CA1 Neurons After TBI

**Figure 3:**
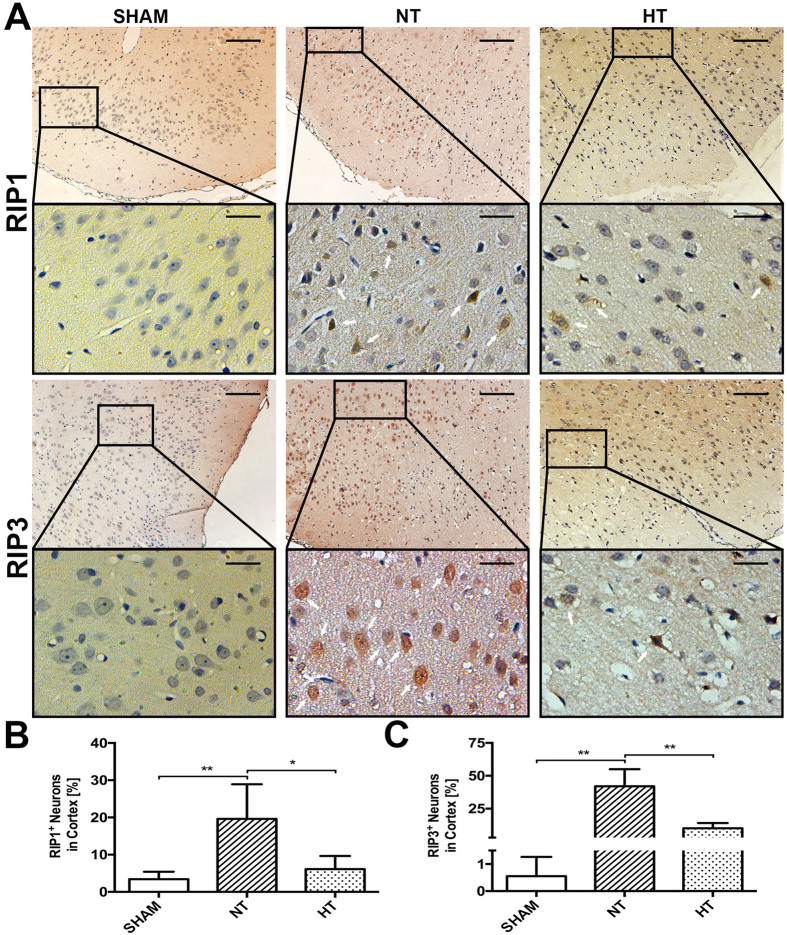
Characterization of RIP1 and RIP3 expression in cortical neurons. Rats were sacrificed at 6 h after TBI. The brains of SHAM, TBI-Normothermia (NT) and TBI-Hypothermia (HT) were obtained. (**A**) Representative photographs of immunostaining for RIP1 and RIP3 in the cortex are shown (white arrows indicate immunoreactivity positive neurons). Original magnification ×100, magnification ×400 for interest area, the scale bars represented 200 μm and 50 μm respectively. (**B**) Statistical analysis of RIP1^+^ and RIP3^+^ neurons in the respective group (n = 6); Data are expressed as mean ± SD. *p < 0.05; **P < 0.01.

**Figure 4:**
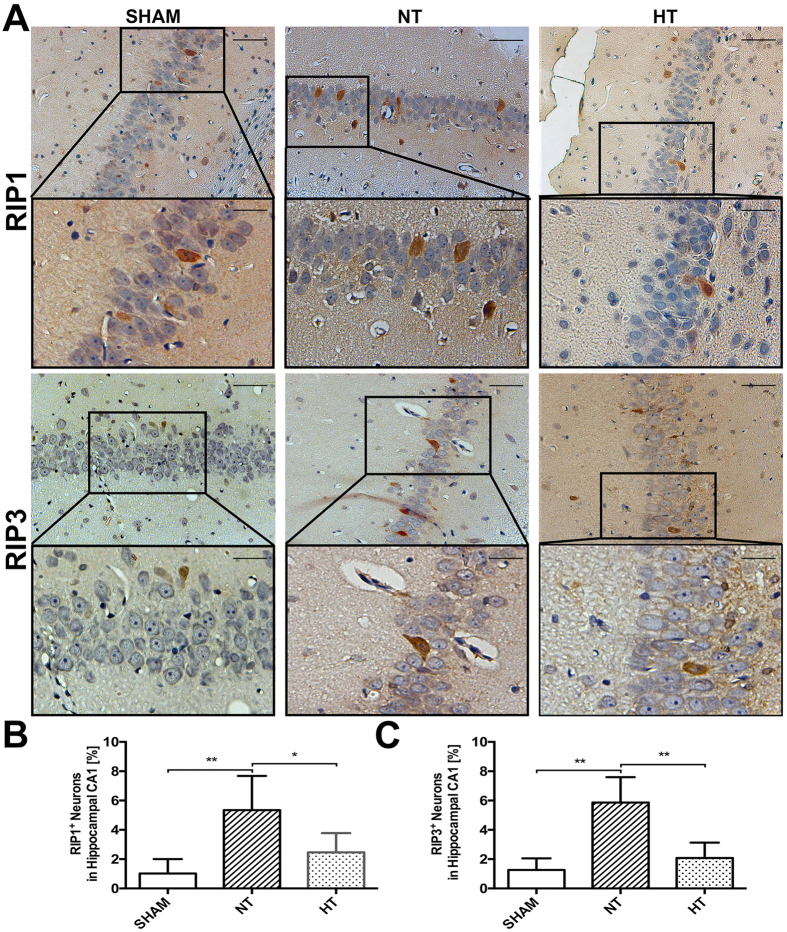
Characterization of RIP1 and RIP3 expression in hippocampal CA1. Rats were sacrificed at 6 h after TBI. The brains of SHAM, TBI-Normothermia (NT) and TBI-Hypothermia (HT) were obtained. (**A**) Representative photographs of immunostaining for RIP1 and RIP3 in the hippocampus CA1 are shown. Original magnification ×200, magnification ×400 for interest area, the scale bars represented 100 μm and 50 μm respectively. (**B**) Statistical analysis of RIP1^+^ and RIP3^+^ neurons in the respective group (n = 6); Data are expressed as mean ± SD. *p < 0.05; **P < 0.01.

In order to detect the cellular effects of hypothermia on RIP1 and RIP3 protein distribution at 6 h after TBI, immunohistochemical analysis was performed in the brain sections of sham and injured (normothermia and hypothermia) animals. Unfortunately, the anti-MLKL antibody could not be used for IHC staining because of nonspecific staining. As shown in Fig. 3, in normothermic animals comparing to the sham-operated, RIP1 and RIP3 immunoreactivity was dramatically increased in a plenty of cytoplasm of neurons in cortex (p < 0.01). In contrast, in the hypothermia group, less RIP1 (p < 0.05) or RIP3 (p < 0.01) positive staining neurons were observed. Additionally, elevated RIP3 expression may coincide with the nuclear translocation of RIP3 as display in several RIP3 stained neurons showing pyknotic nuclei in normothermic groups, which is consistent with the recent study in global cerebral ischemia/reperfusion injury^32^. However, unlike the wide ranged immunoreactivity in cortex, there was only a small but significant number of positive neurons been observed in the CA1 region of hippocampal after TBI compared with sham animals (p < 0.01), and both RIP1 (p < 0.05) and RIP3 (p < 0.01) staining was able to be reduced by hypothermia (Fig. 4).

### Hypothermia Inhibits Brain Tissue Damage in ipsilateral hemisphere after TBI

**Figure 5:**
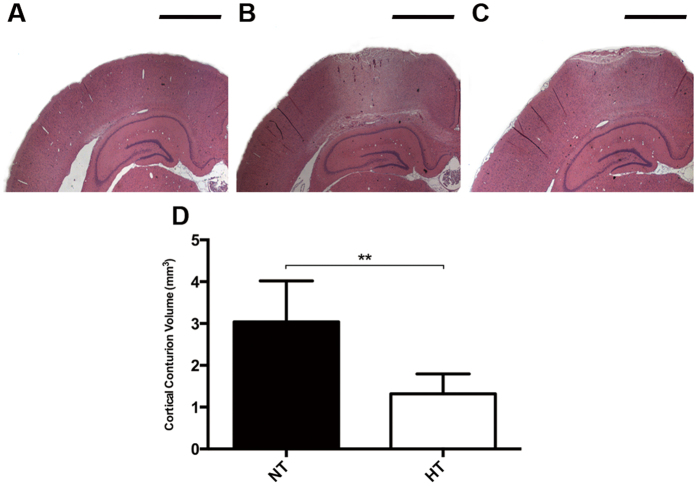
Brain lesion volume in the cortex after TBI. Rats were sacrificed at 6 h after TBI. The brains of SHAM, TBI-Normothermia (NT) and TBI-Hypothermia (HT) were obtained. (**A**–**C**) Representative images of HE staining in ipsilateral hemisphere are shown (n  = 2, 6, 6, respectively). All images were shown at bregma level −4.44 mm. Scale bars (**A**–**C**): 1600 μm. (**D**) Statistical analysis of contusion volume in the NT and HT groups. Data are expressed as mean ± SD. **P < 0.01.

The neuroprotective effect of therapeutic hypothermia was assessed quantifiably by contusion volume in ipsilateral hemisphere. There is no pathology evident seen in sham animals (Fig. 5A). In contrast, all TBI rats had visible, well-demarcated, cortical contusions along the gray/white matter interface (Fig. 5B,C). Animals applied hypothermia treatment after FPI exhibited a greater decrease in contusion volume compared to TBI-normothermic animals at 6 h (Fig. 5D, p < 0.05). Therefore, protection against TBI-induced brain tissue damage is also obtaining in an early phase after application of therapeutic hypothermia compared to previous reports shown similar results at three days^24^.

### Expression of Inflammatory Molecules

**Figure 6:**
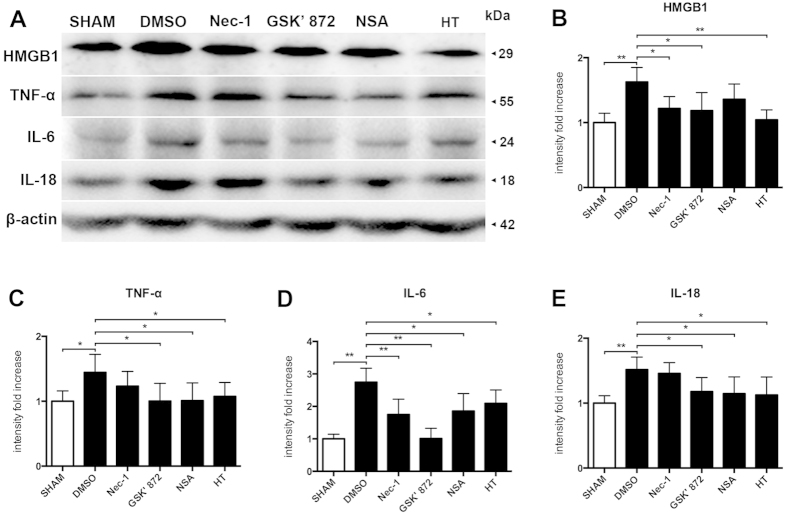
The anti-inflammatory effects targeted necroptosis after TBI. The treatment of hypothermia, DMSO, Nec-1, GSK′ 872 and NSA were performed at 30 minutes after FPI. Rats were sacrificed at 6 h after surgical operation. (**A**) Cortical expressions of HMGB1, TNF-α, IL-6 and IL-18 were assessed by Western blot analysis. β-actin was used as the loading. (**B**–**E**) Quantitative analysis of HMGB1, TNF-α, IL-6 and IL-18 expressions were performed (n = 6 each). Data are expressed as mean ± SD. *p < 0.05, **p < 0.

To analyze the connections between necroptosis and inflammatory responses and the neuroprotective effects of therapeutic hypothermia on TBI model, we examined the expression of inflammation-related molecules on ipsilateral cerebral cortex using Westernbloting. The specific inhibitors of necorptosis, Nec-1, GSK′ 872 and NSA targeted RIP1, RIP3 and MLKL respectively, were administered by icv at 30 minutes after injury (Fig. 6). Increased expression of HMGB1, TNF-α, IL-6 and IL-18 was observed after percussion injury, and all of these molecules was significantly suppressed following treatment with GSK′ 872 and hypothermia. The expression of TNF-α, IL-6 and IL-18 was decreased in the NSA treated groups, and the expression of HMGB1 and IL-6 was also reduced by Nec-1. These data suggest the presence of an induction mechanism mediated by necropotosis after TBI.

## Discussion

Our results first demonstrate that RIP1, RIP3 and MLKL, the critical regulators of necroptosis, transiently increase at an early stage in ipsilateral parietal cerebral cortex following moderate FPI in rats. Further more, the numbers of RIP1- and RIP3-immunoreactive neurons predominantly increase in parietal cerebral cortex at TBI-normothermic animals, while a small but statically significant increased number of positive neurons is also observed in hippocampal CA1. Additionally, we found that nuclear translocation of RIP3 caused by TBI stress is consistent with the observations in experimental cerebral ischemia/reperfusion injury^32^. Most importantly, our data suggested that the TBI-induced increase of necroptosis-associated proteins, contusion volume and expression of inflammatory molecules was able to reduce by the treatment of hypothermia and necroptosis inhibitors in cortex. Taken together, these data demonstrate that necoptosis is involved in the pathologic process of tissue damage and inflammatory response after traumatic brain injury in rat, and the neuroprotective effects of early cooling could benefit from inhibiting necroptosis regulators, which may help to further elucidate the underlying mechanisms regarding the protection of therapeutic hyipothermia on TBI.

For many years, the detailed mechanisms for cell death or neuronal loss after TBI are still unclear, and the reasons could be multifactorial. In the TBI model, apoptosis is one of the major modes of programmed cell death that has been extensively studying, requiring the sequential activation of Bcl-2 and caspase family, eventually resulting in the release of proapoptotic proteins from the mitochondria^33^,^34^. RIP1 was reported to participate in apoptosis when NF-κB driven transcription of cIAP1 and cIAP2 are inhibited during death receptor stimulation^35^. However, Classic apoptosis induced in cells by TNF-α and cycloheximide does not require RIP1, as the deficient of RIP1 in cells does not block apoptosis^35^,^36^. In this study, we show that the level of RIP1 protein and positive neurons increased transiently in cortex following TBI, which therefore underlie the previous researches showing that inhibition of RIP1 by Nec-1 reduced histopathology and improves functional outcome after controlled cortical impact in mice^18^. According to Sutton *et al.*^37^, contusion necrosis was observed in the hours to days (6 h to 30 days) following unilateral controlled cortical impact in rat^37^. Although necrosis has long been thought as an accidental cell death occurring when the cell is suffered an overwhelming stress with subsequent activated inflammasome, it turns out that the molecular mechanisms of pathological cell loss, as primarily reported in ischaemia–reperfusion models, partially overlap with the biochemical cascades mediated necroptosis^4^,^9^.

Necroptosis has been identified to be an alternative type of cell death both in neurons^4^,^38^ as well as in non-neuronal systems^17^,^39^. In most of studies, Nec-1, the potent and effective inhibitor of RIP1, has been approving to rescue cell death and provide neuroprotective effects in certain situations^4^,^18^,^19^. As similar to these findings, we observed that the transient increase of RIP1 in injured cortex was able to prevent by therapeutic hypothermia in an early phase. Although no significant difference of RIP3 and MLKL expression was observed in ipsilateral hippocampus between SHAM- and TBI-group in the experiments of western blotting, the results of immunohistochemistry showed that there was a small but significant increased number of RIP1- and RIP3-staining neurons detected in hippocampal CA1 after TBI, which also observed to be ameliorated by hypothermia. This is in accordance with previous reports demonstrated that early cell death in hippocampal CA1 markedly upregulated at 24 h by TBI could be significantly reduced by post-traumatic mild hypothermia^40^, and that RIP3 protein level was increased in the CA1 region of the hippocampus of rats at 48 h submitted to global cerebral ischemia, while CA3 and DG regions remained constant^38^. These findings implied that the region of hippocampal CA1 might be selectively vulnerable to traumatic brain injury at early stage and that necroptosis may contribute to the neurodegeneration. However, since we did not further separate the hippocampus into CA1, CA3 and DG regions in the present study, we cannot rule out the possibility that proteins regarding necroptosis may upregulate selectively in respective region of hippocampus following TBI. Despite the fact that RIP1 has been approved to involve necroptosis processing and nec-1 made protective effects for cells *in vivo* and *in vitro*^4^,^41^, recent researches demonstrated that RIP1 has important functions in innate immune signaling and DNA damage response and RIP1−/− mice suffer from postnatal lethality^42^. However, activation of RIP3 in cells by TNF-α is able to induce necroptosis with absence of RIP1^43^, while engineered mice expressing catalytically inactive RIP3 promotes lethal RIP1- and caspase-8-dependent apoptosis^44^. Additionally, the mitochondrial protein MLKL has recently been identified as a downstream effector of RIP3 and appears indeed to form oligomers that localize to the plasma membrane and eventually results in failure of the ionic homeostasis with subsequent necroptotic cell death^15^. Hence, RIP3 and MLKL-specific inhibitors, such as GSK′872^45^ and NSA^46^, in combine with pan-caspases inhibitor Z-VAD-fmk might be an attractive therapeutic strategy. Meanwhile, our findings indicate that therapeutic hypothermia inhibits RIP1, RIP3 and MLKL protein expression in parietal cortex and the immunostaining of RIP1 and RIP3 in hippocampal CA1 after TBI. Since early studies have attributed the protective effects of hypothermia at acute “early” phase (minutes to hours) to reducing the cerebral metabolic rates of glucose and oxygen, inhibiting excitotoxicity, early gene expression and stress response as well as preventing apoptotic death^47^, the effects of moderate hypothermia on necroptosis signal pathway after TBI may be accounted for preventing TNFR1 signaling, a canonical pathway, via early activation of the JNK signaling pathway and caspase-3, to induce necroptosis initially^48^. Moreover, in our view, hypothermia may potentially reduce the effects after activated MLKL translocate to the membrane, which deserved a further study.

An important finding in our study was that hypothermia treatment remarkably reduced posttraumatic lesion size after FPI. This finding is in consistent with previous study demonstrated a similar outcome at there days post-injury^24^, and suggest that tissue damage is just happen at 6 h after TBI and the protective effects of therapeutic hypothermia for TBI perform at an early phase. Since a plenty of works have been done for studying the effects of hypothermia on behavioral outcomes in rodents following TBI^49^, we did not further evaluate the neurological scores in the present study. On the basis of prior studies showing that RIP3-dependent necroptosis of retinal pigment epithelial cells induce inflammation in a mouse model of retinal degeneration while RIP3 deficiency decreased levels of pro-inflammatory cytokines (such as TNF-α and IL-6) in the retina, and attenuated intravitreal release of HMGB1^50^, RIP3- and MLKL-dependent necrosis exacerbate tissue injury and inflammation in the model of acute pancreatitis^51^,^52^, whereas RIP3 deficiency is protective in a mouse model of Gaucher’s disease and ameliorated ischaemia-reperfusion-induced injury and inflammation^53^,^54^, we anticipated that necroptosis would play a proinflammatory role after FPI. Compared with DMSO group, treatment with hypothermia and GSK′ 872 significantly reduced levels of HMGB1, TNF-α, IL-6 and IL-18 in the cortex after injury, as well as Nec-1 and NSA also partially decreased the expression of pro-inflammatory cytokines. Inconsistent with previous studies indicated that the level of HMGB1, TNF-α, IL-6 and IL-18 is increased after TBI^55^,^56^,^57^. These data suggest that necroptotic mechanisms contribute to inflammatory responses after TBI. Moreover, hypothermia ameliorated the release of pro-inflammatory cytokines after TBI, a beneficial effect that might be attribute to inhibited necroptosis signaling in this model, as the upstream regulators of necroptosis, TNFR1 signaling is able to mediate by post-traumatic hypothermia^48^.

In summary, these data suggest that necroptosis, a novel form of programmed cell death triggered by TNFR1, may play a significant role in tissue damage and inflammatory responses after FPI in rat. This study provides proof of principle that the interventions to control or inhibit necroptosis at an early stage after the primary insult that could have beneficial effects for TBI patients.

## Additional Information

**How to cite this article**: Liu, T. *et al.* Therapeutic hypothermia attenuates tissue damage and cytokine expression after traumatic brain injury by inhibiting necroptosis in the rat. *Sci. Rep.* **6**, 24547; doi: 10.1038/srep24547 (2016).

## Figures and Tables

**Table 1:** Data of physiology.

	Pre-trauma 15 min	Post-trauma 15 min	Post-trauma 4 h
SHAM			
pH	7.41 ± 0.04	7.45 ± 0.03	7.43 ± 0.07
PCO_2_ (mmHg)	39.6 ± 2.1	38.2 ± 2.2	38.8 ± 1.9
PO_2_ (mmHg)	123.1 ± 3.9	130.9 ± 5.5	139.9 ± 6.2
MAP (mm Hg)	100.3 ± 4.4	101.4 ± 3.9	104.9 ± 2.6
Brain temperature (°C)	36.97 ± 0.11	37.04 ± 0.09	36.99 ± 0.01
Rectal temperature (°C)	36.91 ± 0.03	36.81 ± 0.08	36.92 ± 0.08
Normothermia			
pH	7.43 ± 0.02	7.40 ± 0.01	7.46 ± 0.04
PCO_2_ (mmHg)	39.9 ± 1.3	38.4 ± 1.9	38.8 ± 2.0
PO2 (mmHg)	125.4 ± 2.5	128.8 ± 3.3	129.1 ± 7.5
MAP (mm Hg)	103.2 ± 3.1	104.7 ± 2.2	99.1 ± 3.1
Brain temperature (°C)	36.95 ± 0.02	37.11 ± 0.01	37.00 ± 0.01
Rectal temperature (°C)	36.78 ± 0.02	36.32 ± 0.16	36.78 ± 0.11
Hypothermia			
pH	7.41 ± 0.01	7.45 ± 0.07	7.46 ± 0.04
PCO_2_ (mmHg)	39.0 ± 1.4	39.3 ± 2.6	39.7 ± 1.8
PO2 (mmHg)	136.9 ± 5.5	120.9 ± 4.5	129.9 ± 2.5
MAP (mm Hg)	101.1 ± 1.1	100.7 ± 4.9	102.3 ± 1.8
Brain temperature (°C)	36.93 ± 0.02	36.82 ± 0.07	33.11 ± 0.02*
Rectal temperature (°C)	36.98 ± 0.01	37.01 ± 0.02	33.05 ± 0.01*

MAP, mean arterial pressure. Values are expressed as means ± SD. The physiological values were all within normal ranges. Significant differences were detected between sham and normothermic groups compared with hypothermia for postinjury temperature readings, *P < 0.05.
